# Moyamoya Syndrome in a 32-Year-Old Male With Sickle Cell Anemia

**DOI:** 10.7759/cureus.10001

**Published:** 2020-08-24

**Authors:** Mariam Yamani, Elaf F Obaid, Amr H Hemida

**Affiliations:** 1 Medicine, Umm Al-Qura University, Mecca, SAU; 2 Internal Medicine, Al Noor Specialist Hospital, Mecca, SAU

**Keywords:** moyamoya disease, moyamoya syndrome, sickle cell disease, vasculopathy

## Abstract

Moyamoya disease (MMD) is an unusual vasculopathy in which the blood vessels of the brain are occluded, resulting in thrombosis. When MMD occurs in association with an underlying pathology, it is known as moyamoya syndrome. The association of moyamoya syndrome with sickle cell disease is uncommon. Herein, we report a case of moyamoya syndrome in a 32-year-old male adult patient.

## Introduction

Moyamoya disease (MMD) is an uncommon progressive vasculopathy that affects the cerebral arteries. It is characterized by stenotic changes involving the brain blood vessels, most commonly the internal carotid artery (ICA). Characteristically, MMD is associated with a pathological formation of a new blood vessel known as ‘moyamoya vascular’, which is a Japanese term that denotes a cloud of smoke. This term was first introduced in 1969 to describe the radiological findings of these distinct blood vessels in cerebral angiograms [1-4].

Epidemiologically, MMD occurs mostly in children and rarely in adults [5]. Additionally, MMD has a high incidence rate in East Asian countries, predominantly Korea and Japan (three cases per 100,000 of the pediatric population). In Europe, the incidence rate of MMD is comparatively ten times lower [5]. In Saudi Arabia, the incidence rate of MMD is not well-defined; however, it is extremely rare with roughly less than 20 reported cases. They were mostly scattered case reports [6,7] and small-sized case series involving pediatric patients [8-11].

MMD refers to the disease itself without a predisposing condition. However, moyamoya syndrome (MMS) refers to MMD in the presence of a predisposing condition, such as neurofibromatosis, systemic lupus erythematosus, tuberous sclerosis and sickle cell disease (SCD) [1,12]. The association of SCD with MMS is uncommon [13]. To the best of our knowledge, less than 200 cases have been reported around the world [14]. Specifically, in Saudi Arabia, only three cases of MMS in association with SCD have been reported and all of them involved pediatric patients [8,9]. Herein, we report the first case of MMS in a sickler Saudi Arabian adult patient.

## Case presentation

A 32-year-old Saudi Arabian male sickler patient presented to the emergency department with a six-hour history of sudden and continuous shortness of breath. The history was obtained from the mother. The patient’s current presentation was associated with agitation and a sudden change in behavior. The patient was shouting and refusing to communicate with doctors. The mother denied any history of orthopnea, cough or fever during the patient’s current presentation. Past medical history was remarkable for SCD since childhood with infrequent hospital visits. Socially, the patient was single, non-smoker and living with his family.

On general examination, the patient looked ill, anxious and sitting on the bed lying forward connected to an oxygen mask. The body mass index (BMI) was 18.5 kg/m^2^. Vital signs showed blood pressure: 125/80 mmHg, pulse: 110 beats/min, respiratory rate: 28 breaths/min, temperature: 39 degrees Celsius and oxygen saturation: 87% on room air and 94% on 5 liters of oxygen. Respiratory and cardiovascular examinations were normal. Hemoglobin electrophoresis showed a hemoglobin S (Hb S) level of 78%. Laboratory testing for D-dimer showed 12 ng/L (normal range is < 0.5 ng/L). Pulmonary embolism was suspected and chest spiral computed tomography (CT) scan showed bilateral consolidation without acute pulmonary embolism. In consideration of the fever and agitation, the patient received ceftriaxone, vancomycin and diazepam.

**Figure 1:**
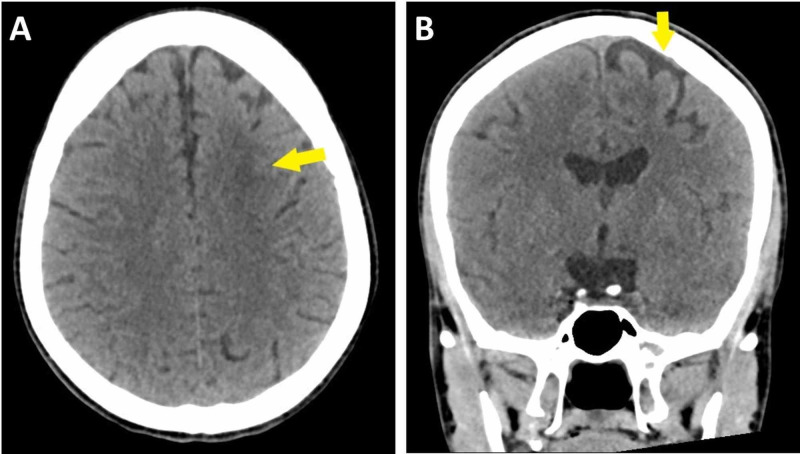
Axial computed tomography scan of the head without contrast showing left frontal subcortical deep white matter hypodensity, most likely representing chronic watershed infarction (A). Coronal computed tomography scan of the head without contrast showing left frontal cortical gray matter hypodensity, most likely representing cerebral atrophy (B).

**Figure 2:**
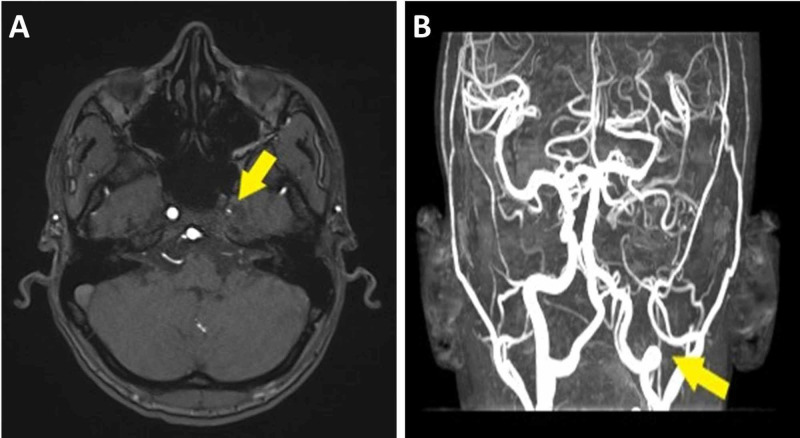
Magnetic resonance angiography showing severely hypoplastic left internal carotid artery (A) and abnormal tortuous blood vessels around circle of Willis (B).

CT scan of the head without contrast showed left frontal subcortical deep white matter hypodensity, most likely representing chronic watershed infarction. Moreover, there was left frontal cortical gray matter hypodensity, most likely representing cerebral atrophy (Figure 1). There were no signs of acute hemorrhage or ischemic infarction. Magnetic resonance angiography (MRA) showed severely hypoplastic left ICA and abnormal tortuous collateral vessels around the circle of Willis (Figure 2). No arteriovenous malformation or aneurysm was noted.

Based on the clinical and radiological findings, the final differential diagnosis was suggestive of a progressive vascular occlusive process which could be seen in MMD. Hematology team was consulted for further evaluation. The patient received a blood transfusion three times daily for 16 days. The patient dramatically improved and returned to his regular behavior. The patient was discharged home on aspirin 81 mg daily. The patient was seen four weeks later at the neurology clinic and he was doing well.

## Discussion

The exact pathogenesis of MMD is not clearly understood [13]. Risk factors for MMD include East Asian genetics, pediatric age group, female gender and family history of MMD [1,4,12]. Common underlying conditions associated with MMS include neurofibromatosis, systemic lupus erythematosus, tuberous sclerosis, periarteritis nodosa, mesial temporal sclerosis, thyrotoxicosis, Down’s syndrome and previous radiation therapy [1,4,12]. The association of MMS with SCD is not common [14]. MMD typically involves children in the first decade of life (average is nine years of age) and rarely adults (around 30-40 years of age) [5,15]. Children most often present to clinical attention with ischemic strokes or seizures. On the other hand, adults most commonly present to clinical attention with hemorrhagic strokes [4]. Epidemiological studies confirm that MMD/MMS is most frequent in East Asian countries and very occasional in Western countries [4]. Our patient was an adult of a Middle-eastern origin and presented with shortness of breath and agitation.

Diagnostic criteria for definitive MMD include occlusive changes at the ICA bilaterally or unilaterally as well as abnormal collateral blood vessels based on radiological imaging [4,5]. Catheter angiography is the definitive diagnostic test; however, it is relatively invasive. MRA and CT angiography are the best and practical diagnostic tests. This is because they are less invasive and can detect distal steno-occlusion in ICA and/or middle cerebral artery. Nonetheless, they are less sensitive to detect basal collaterals [4,5,16].

Medical management of MMS with SCD includes blood transfusions which can reduce the clinical symptoms and complications such as strokes, transient ischemic attacks and seizures. Nevertheless, recent studies imply chronic transfusions do not prevent the disease progression and that revascularization surgeries are actually more effective in the long-run, especially before the onset of neurological deficits [1,12,14,17,18].

The natural history of MMD is wide-ranging. Disease progression can occur slowly with occasional stroke-like events or rapidly with advanced neurologic decline [19]. Even among asymptomatic patients, the rate of disease progression over time is high without surgical management [20]. The neurologic status at the time of management is the most important factor that predicts long-term outcomes in patients with MMD [19]. Additionally, irrespective of treatment, once a major neurologic incident occurs, such as stroke or bleeding, the patient may experience a permanent loss of function with poor prognosis. Thus, early diagnosis of MMD along with prompt surgical intervention is central to achieving better disease outcomes.

## Conclusions

In conclusion, MMD is an uncommon progressive vasculopathy that mostly affects cerebral arteries. Although rare, however, MMS should be considered in the differential diagnosis in adults with SCD and presenting with shortness of breath. Brain angiography can aid in establishing the definitive diagnosis. Revascularization surgery is preferred over medical treatment in patients with MMS and SCD.
